# mTORC2 affects the maintenance of the muscle stem cell pool

**DOI:** 10.1186/s13395-019-0217-y

**Published:** 2019-12-02

**Authors:** Nathalie Rion, Perrine Castets, Shuo Lin, Leonie Enderle, Judith R. Reinhard, Markus A. Rüegg

**Affiliations:** 1 Biozentrum, University of Basel, CH-4056 Basel, Switzerland; 2 Department PHYM, Centre Médical Universitaire de Genève, Geneva, Switzerland; 3 Toronto Recombinant Antibody Centre/The Donnelly Centre, University of Toronto, M5G 1 L6, Toronto, ON Canada

**Keywords:** mTORC2, Rictor, Muscle regeneration, Muscle stem cells

## Abstract

**Background:**

The mammalian target of rapamycin complex 2 (mTORC2), containing the essential protein rictor, regulates cellular metabolism and cytoskeletal organization by phosphorylating protein kinases, such as PKB/Akt, PKC, and SGK. Inactivation of mTORC2 signaling in adult skeletal muscle affects its metabolism, but not muscle morphology and function. However, the role of mTORC2 in adult muscle stem cells (MuSCs) has not been investigated.

**Method:**

Using histological, biochemical, and molecular biological methods, we characterized the muscle phenotype of mice depleted for rictor in the Myf5-lineage (RImyfKO) and of mice depleted for rictor in skeletal muscle fibers (RImKO). The proliferative and myogenic potential of MuSCs was analyzed upon cardiotoxin-induced injury in vivo and in isolated myofibers in vitro.

**Results:**

Skeletal muscle of young and 14-month-old RImyfKO mice appeared normal in composition and function. MuSCs from young RImyfKO mice exhibited a similar capacity to proliferate, differentiate, and fuse as controls. In contrast, the number of MuSCs was lower in young RImyfKO mice than in controls after two consecutive rounds of cardiotoxin-induced muscle regeneration. Similarly, the number of MuSCs in RImyfKO mice decreased with age, which correlated with a decline in the regenerative capacity of mutant muscle. Interestingly, reduction in the number of MuSCs was also observed in 14-month-old RImKO muscle.

**Conclusions:**

Our study shows that mTORC2 signaling is dispensable for myofiber formation, but contributes to the homeostasis of MuSCs. Loss of mTORC2 does not affect their myogenic function, but impairs the replenishment of MuSCs after repeated injuries and their maintenance during aging. These results point to an important role of mTORC2 signaling in MuSC for muscle homeostasis.

## Background

Myogenesis is the process in which progenitors, characterized by expression of the paired box protein 3 and 7 (Pax3/Pax7), differentiate to form skeletal muscle fibers. Individual steps of myogenesis are controlled by the sequential expression of myogenic basic-helix-loop-helix (bHLH) transcription factors Myf5, MyoD, Myogenin, and Mrf4 [1]. Myogenesis is responsible for the formation of skeletal muscle fibers both, during embryogenesis and after injury in the adult. The progenitor cells that undergo the myogenic process in adult skeletal muscle fibers are muscle stem cells (MuSCs), also called satellite cells [2]. MuSCs express several markers, including Pax7, and the vast majority of MuSCs transiently express *Myf5* during embryonic myogenesis or start expressing *Myf5* upon activation of MuSCs after injury [3].

The mammalian (or mechanistic) target of rapamycin (mTOR) participates in two structurally and functionally distinct complexes, called mTOR complex 1 (mTORC1) and mTOR complex 2 (mTORC2). mTORC1, containing the protein raptor, regulates cell growth by controlling the balance between protein synthesis and protein degradation [4]. In contrast, mTORC2 with its essential component rictor, regulates ion transport and cell survival by phosphorylating the serine/threonine protein kinase 1 (SGK1, at Serine 422) [5], as well as actin cytoskeleton organization via the phosphorylation of protein kinase C α (PKCα, at Serine 657) [6, 7]. In recent years, mTORC2 has also been shown to regulate lipogenesis and glucose homeostasis in insulin-stimulated tissues (e.g., liver) by phosphorylating (at Serine 473) its best-described target protein kinase B (PKB/Akt) [8–10]. Genetic inactivation of mTORC2 in skeletal muscle fibers via deletion of *Rictor*, using human skeletal actin (HSA)-Cre or muscle creatine kinase (MCK)-Cre, does not result in an overt muscle phenotype [11] but impairs insulin-stimulated glucose transport and increases glycogen synthase activity [12]. Moreover, whole-body metabolism of mutant mice is perturbed due to a re-partitioning of lean to fat mass and an increase in intramyocellular triglycerides, which results in the preferred usage of fat as an energy substrate [13]. In cultured C2C12 myoblasts, knock-down of *Rictor* by siRNA blocks terminal differentiation through PKB/Akt and the Rho-associated kinase 1 [14], suggesting a role of mTORC2 in myogenesis. In mice, deletion of *Rictor* using *Myf5*-Cre (i.e., depleting rictor in progenitor cells that give rise to myoblasts and brown adipocytes) revealed an important function of mTORC2 for brown adipocyte differentiation and growth [15]. However, embryonic myogenesis was largely unaffected, which was in stark contrast to *Myf5*-Cre-driven depletion of raptor (inactivation of mTORC1), which caused perinatal death of the mice [16].

Here, we focused on the characterization of *Myf5*-Cre-driven deletion of *Rictor* in adult mice and show that mTORC2 signaling is dispensable for muscle function and muscle regeneration in 5-month-old mice. Consistent with these results, the proliferative and myogenic function of MuSCs in single muscle fibers in vitro was not affected. However, we observe an impairment in MuSC renewal upon repeated muscle injury and in MuSC maintenance during natural aging. Hence, our work unravels a novel role of mTORC2 for muscle homeostasis.

## Methods

### Mice

RImyfKO mice were obtained by crossing *Rictor*-floxed mice [11] with mice expressing *Cre recombinase* from the *Myf5* locus [17] (obtained from Jackson Laboratories). Genotyping for the floxed *Rictor* and the *Myf5-Cre* alleles was performed as described previously [11, 17]. Generation and genotyping of HSA-driven deletion of *Rictor*, called RImKO mice, was described previously [11]. Females or males were used. No gender-specific differences were observed except where indicated. In vitro force measurements of the extensor digitorum longus (EDL) and soleus (Sol) muscles were performed as described [11]. Body composition was analyzed by magnetic resonance in conscious, immobilized mice using the EchoMRI-100H body composition analyzer (EchoMRI). All mice were kept under standard conditions with a fixed 12-h dark-light cycle and free access to food and water. All animal studies were approved by the veterinary office of the Canton of Basel, in accordance to Swiss regulations.

### Quantitative PCR

Total RNA was extracted from frozen TA muscle with the SV Total RNA Isolation System (Promega) and cDNA prepared using the iScript cDNA Synthesis Kit (Bio-Rad) following the manufacturer’s instructions. Real-time PCR was performed with FastStart Essential DNA Green Master (Roche) using the LightCycler 480 II (Roche). *Gapdh* was used as reference gene. The following primers were used: *Pax7* s: GAG GTG ACA GGA GGC AGA AG, *Pax7* as: AGC TGC CAG CAA GAT GGT AT, *Myod1* s: CAT TCC AAC CCA CAG AAC CT, *Myod1* as: TGC TGT CTC AAA GGA GCA GA, *Myog* s: GTG AAT GCA ACT CCC ACA G, *Myog* as: AGC AAA TGA TCT CCT GGG T, *Gapdh* s: ACC CAG AAG ACT GTG GAT GG, and *Gapdh* as: GGA TGC AGG GAT GAT GTT CT.

### Muscle injury and regeneration

Five- or 14-month-old mice were anesthetized with Ketamine (111 mg/kg, Ketalar) and Xylazine (22 mg/kg, Rompun) by intraperitoneal injection. Tibialis anterior (TA) was unilaterally injected with 6.7 μg cardiotoxin (Ctx; Latoxan) to provoke muscle degeneration. Analgesia was provided by application of 0.1 mg/kg Buprenorphine, twice a day, for at least 3 days. Subsequent injuries were induced only after a 30-day waiting period. The injured, regenerating TA and the attached EDL muscle, as well as the uninjured, contralateral muscles were harvested 7- or 15-day post-injury.

### Single myofiber isolation and culture

Single muscle fibers were isolated from EDL muscle of 5-month-old mice by enzymatic digestion and trituration as previously described [18]. In brief, EDL was digested with 1 mg/ml Collagenase A (Roche) in DMEM Glutamax (Gibco), 1% penicillin-streptomycin (Thermo Fisher Scientific) for 1.5 h at 37 °C and triturated into single myofibers. Fibers were cultured in DMEM Glutamax supplemented with 1% penicillin and streptomycin, 10% horse serum, and 1% chicken embryo extract. The cells were fixed with 4% paraformaldehyde (PFA). For immunostaining, fibers were permeabilized with PBS, 0.5% Triton-X100, and blocked for 30 min with PBS supplemented with 10% horse serum, 10% goat serum, and 0.35% Carrageenan (Sigma). Primary antibodies were incubated overnight at 4 °C. Fibers were washed with PBS, 0.025% Tween-20, and incubated with secondary antibodies for 1.5 h. Myofibers were then mounted on slides coated with 84% acetone, 16% (3-aminopropyl)triethoxysilane (Sigma), with Vectashield Dapi (Vector).

### Histology and immunofluorescence staining

After dissection, muscles were frozen in nitrogen-cooled isopentane. Consecutive, frozen cross-sections of 8 μm of thickness were used for standard histological staining. Hematoxylin and eosin staining (Merck) was followed by sequential dehydration with 70%, 90%, and 100% ethanol, and 100% xylene. Picrosirius red (1 mg/ml in 1.3% aqueous solution of picric acid, Sigma) was applied for 1 h, followed by washing in 0.5% acidic water for 30 min. After dehydration in 100% ethanol, the slides were cleared in xylene. For Oil red O staining, sections were fixed with 4% PFA for 1 h and stained with 5 mg/ml Oil red O, 60% triethyl-phosphate (Sigma) for 30 min. The sections were washed with water and mounted in 10% glycerol.

For immunohistochemistry, cryosections were fixed with 4% PFA for 6 min, washed in PBS pH 7.4, 0.1 M glycine, and permeabilized with pre-cooled methanol for 6 min. Antigen retrieval was performed by warming the sections in 0.01 M citric acid. Sections were blocked in 3% IgG-free BSA (Jackson Immuno Research Laboratories), supplemented with 0.05 mg/ml AffiniPure Mouse IgG, Fab Fragment (Jackson Immuno Research Laboratories). Primary antibodies were incubated overnight at 4 °C. Sections were subsequently washed and incubated with the appropriate fluorescent secondary antibodies for 1.5 h at room temperature. After washing with PBS, samples were mounted with Vectashield Dapi (Vector laboratories). DAPI staining was used to assure that the Pax7 signal was specific. For myosin heavy chain (MHC) II and embryonic myosin heavy chain (embMHC) staining, PFA fixation was omitted.

To quantify embMHC-positive fibers, cross-sections of TA muscles were stained for embMHC. Sections were stained against laminin and with DAPI. Seven images (512 × 512 pixels) were recorded from each section using Zeiss LSM700 point scanning confocal microscope with × 20 objective. To count the number of embMHC-positive fibers, acquisition parameters were set the same for all sections. The maximal intensity projections of images were analyzed using Image J software. EmbMHC-positive fibers were identified by setting the same intensity-threshold for all images.

### Antibodies

Rabbit polyclonal antibodies were as follows: MyoD c-20 (sc-304) from Santa Cruz; laminin (ab11575) from Abcam; mouse monoclonal antibodies were as follows: Pax7, myosin heavy chain (human fast fibers; A4.74) and myosin heavy chain embryonic (A1.652) from Developmental Studies Hybridoma Bank (DSHB). Rat monoclonal antibodies against laminin-α2 (ab11576) and rabbit polyclonal antibodies against laminin (ab11575) were from Abcam.

### Statistical analyses

For muscle fiber size quantification, images were acquired using a Leica DM5000B fluorescence microscope with × 10 objective, a digital camera (F-View; Olympus Soft Imaging Solutions GmbH) and the analySIS software (Soft Imaging System). The minimum distance of parallel tangents at opposing particle borders (minimal feret’s diameter) of muscle fibers from the entire TA and EDL muscles or from random fields of the muscles, was measured with the analySIS software as described [19]. For fiber size quantification after Ctx-induced injury, only centronucleated fibers (i.e., regenerating) were included in the quantification. All experiments were performed using a minimum of 3 independent biological samples indicated by the n-number (*n*). In all graphs, data are represented as the mean value and the respective standard error of the mean (SEM). Student’s *t* test and Sidak’s multiple comparisons test were employed to evaluate statistical significance, and *p* values lower than 0.05 were considered statistically significant.

## Results

### The role of mTORC2 in muscle fiber development and growth

**Fig. 1:**
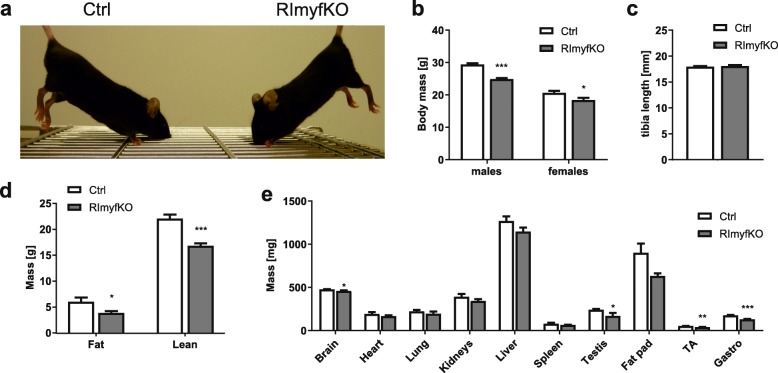
Depletion of rictor in the Myf5-lineage affects whole-body growth. **a** Picture of Ctrl (*Myf5*^*+/+*^*; Rictor*^*fl/fl*^) and RImyfKO (*Myf5*^*+/Cre*^*; Rictor*^*fl/fl*^) mice at the age of 5 months. **b** The body mass of Ctrl and RImyfKO mice was measured at 5 months of age (*n* = 7–9). **c** The tibia length of Ctrl and RImyfKO mice was measured at 5 months of age (*n* = 4–6). **d** The fat and lean mass of 5-month-old Ctrl and RImyfKO mice was analyzed by EchoMRI (*n* = 7). **e** The mass of the organs indicated was measured in 5-month-old Ctrl and RImyfKO mice (*n* = 4–6). Data represent mean ± SEM. *p < 0.05, **p < 0.01, ***p < 0.001, Student’s *t* test

Previous reports have documented that mice in which *Rictor* was deleted in muscle precursor cells (using *Myf5*-Cre) or in skeletal muscle fibers do not show an overt phenotype at young age [11, 12, 15, 16]. When we analyzed RImyfKO (for Rictor myf5 knockout; *Myf5*^*Cre/+*^; *Rictor*^*fl*/*fl*^) more closely, we found that mutant mice weighed significantly less than their littermate controls (Ctrl; *Myf5*^*+/+*^*; Rictor*^*fl/fl*^) at the age of 5 months (Fig. 1a, b), while tibia length was the same (Fig. 1c). The lower body weight was based on the reduction of both, the fat and lean mass (Fig. 1d). Most organs in RImyfKO mice also tended to be lighter but significance was only reached for the brain, testis, and the two muscles examined, the tibialis anterior (TA) and the gastrocnemius (Gastro) (Fig. 1e).

**Fig. 2:**
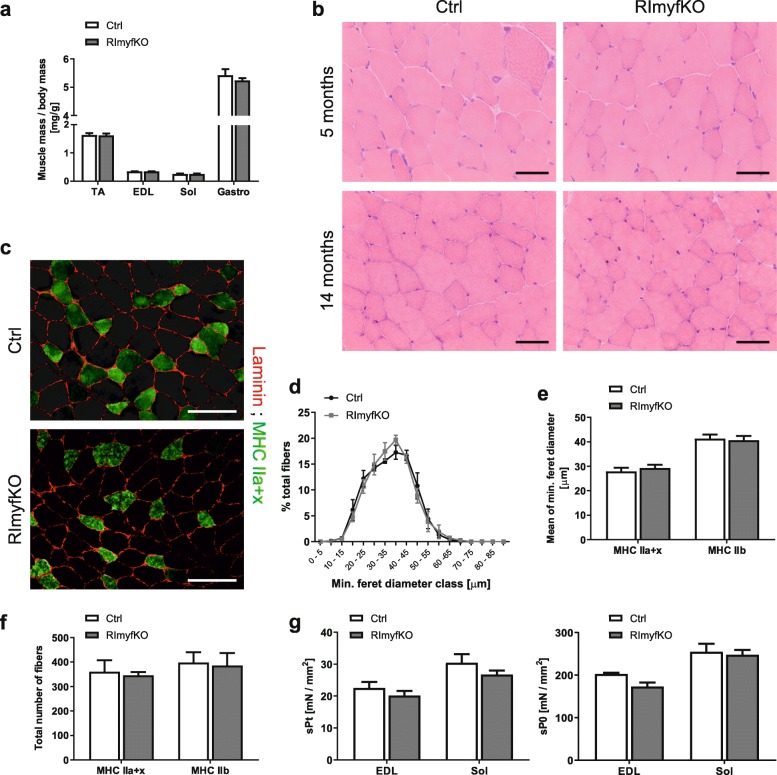
Depletion of rictor does not affect muscle structure and force. **a** Muscle mass, normalized to body mass, of tibialis anterior (TA), extensor digitorum longus (EDL), soleus (Sol), and gastrocnemius (Gastro) muscles from 5-month-old male RImyfKO mice and control (Ctrl) littermates (*n* = 4-6). **b** Hematoxylin and eosin (H&E) coloration of TA cross-sections of 5- and 14-month-old Ctrl and RImyfKO mice. No alteration in muscle histology and fiber organization was detected in RImyfKO muscle. Scale bar, 50 μm. **c** Immunostaining against laminin (red) and myosin heavy chain IIa + *x* (green) of EDL cross-sections from 14-month-old Ctrl and RImyfKO mice. Type IIa fibers are labeled in bright green, type IIx in light green and IIb fibers are unstained. Scale bar, 100 μm. **d**–**f** Fiber size distribution (**d**), mean of minimum feret diameter (**e**), and total number of fibers (**f**) were analyzed in EDL muscle of 14-month-old Ctrl and RImyfKO mice (*n* = 3). **g** Specific twitch force (sPt) and specific tetanic force (sP0) of EDL and Sol muscles from 14-month-old Ctrl and RImyfKO mice (*n* = 4–7). Data represent mean ± SEM. Student’s *t* test

When mass of individual muscles was normalized to body mass, all the muscles of RImyfKO mice were not significantly lighter than those of control mice (Fig. 2a). We also did not detect histological differences in TA muscle from 5- and 14-month-old RImyfKO mice compared to controls (Fig. 2b). Fiber type composition, fiber size distribution, and fiber number were also unchanged (Fig. 2c–f). Ex vivo force measurements of extensor digitorum longus (EDL) and soleus muscles from 14-month-old RImyfKO or control mice showed no difference in the specific twitch (sPt) and specific tetanic (sP0) force, compared to controls (Fig. 2 g). Thus, deletion of *Rictor* in *Myf5*-expressing cells does not affect the formation, the growth, and the force of skeletal muscle.

### mTORC2 signaling is required to maintain the satellite cell pool in adult muscle

**Fig. 3:**
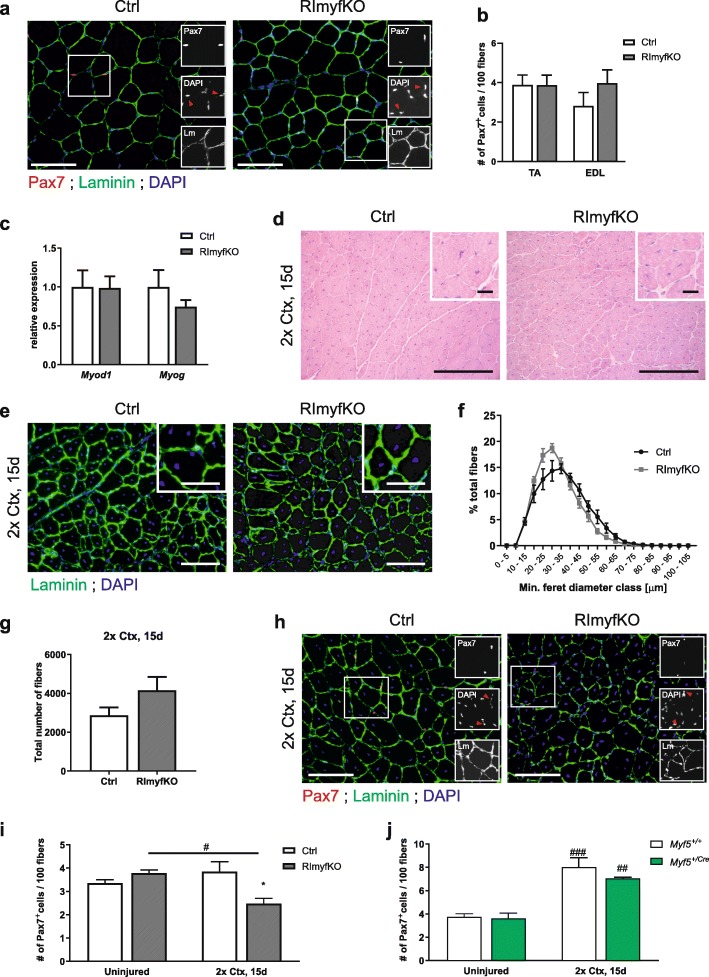
MuSC number after repeated injury is reduced in RImyfKO muscle. **a** Merged and single (insets) staining against Pax7 (red), laminin (green), and for DAPI (blue) of muscle cross-sections from 5-month-old Ctrl and RImyfKO mice. Red arrowheads in insets indicate DAPI-positive nuclei that are also Pax7-positive. Scale bar, 100 μm. **b** The number of Pax7-positive cells per 100 myofibers was counted in TA and EDL muscles from 5-month-old Ctrl and RImyfKO mice (*n* = 4–5). **c** Relative expression of transcripts encoding MyoD and MyoG in TA muscle from 5-month-old Ctrl and RImyfKO mice, normalized to *Gapdh* (*n* = 4). **d**–**j** Muscle regeneration was studied by two rounds of injections of cardiotoxin (Ctx) into TA muscle, 15 days post-injury (2x Ctx, 15 days). **d** H&E staining of TA muscle. Muscle fibers are regenerating irrespective of the genotype as indicated by the centralized nuclei. Scale bar, 100 μm and 10 μm (inset). **e** Immunostaining against laminin (green) and DAPI staining of TA cross-sections. Scale bar, 100 μm and 50 μm (inset). **f** Fiber size distribution and **g** total number of myofibers (*n* = 3). **h** Merged and single (insets) staining against Pax7 (red), laminin (green), and for DAPI (blue) of muscle cross-sections. Red arrowheads in insets indicate DAPI-positive nuclei that are also Pax7-positive. Scale bar, 100 μm. **i** Quantification of the number of Pax7-positive cells/100 myofibers of uninjured and injured muscle (*n* = 4). **j** Quantification of the number of Pax7-positive cells/100 myofibers in uninjured and injured muscle from *Myf5*^*+/+*^ and *Myf5*^*+/Cre*^ mice (*n* = 4–5). The difference in the absolute number of Pax7-positive cells might be due to the different genetic background of the mice. Data represent mean ± SEM. *p < 0.05, Student’s *t* test (difference between genotypes). ^#^*p* < 0.05, ^*##*^*p* < 0.01, ^###^*p* < 0.001, Sidak’s multiple comparisons test (difference between uninjured and injured muscles)

As Myf5-driven Cre expression also causes recombination of floxed genes in most MuSCs [3, 20–22], we next analyzed them in RImyfKO muscle under homeostatic and challenging conditions. In 5-month-old RImyfKO mice, the number of Pax7-positive MuSCs in TA and EDL did not differ from controls (Fig. 3a, b). Moreover, transcript levels coding for MyoD and MyoG were the same in TA muscle from RImyfKO and control mice (Fig. 3c). To challenge MuSCs, we next triggered muscle regeneration by the injection of cardiotoxin (Ctx). We previously showed that muscle regeneration after a single Ctx-induced injury was the same in RImyfKO and control mice [16]. Therefore, we applied a second injury and examined muscles 15-day post-injury. Regeneration after the second injury was very similar in RImyfKO and control mice (Fig. 3d–g). These experiments show that rictor-depleted MuSCs have the same proliferation and differentiation potential as control cells, consistent with results obtained in cultured primary myoblasts [16]. However, we noticed that the number of Pax7-positive cells was significantly reduced in RImyfKO mice after the second round of regeneration (Fig. 3 h, i). The number of MuSCs in RImyfKO mice also tended to be lower when calculating the number of MuSCs per area (Ctrl 10.22 ± 0.69; RImyfKO 8.14 ± 0.53 Pax7^+^ cells/mm^2^; mean ± SEM; *n* = 4; *p* = 0.054). This difference was not caused by the deletion of one *Myf5* allele in RImyfKO mice, since 5-month-old *Myf5*^*+/*Cre^ mice did not show any difference in the number of Pax7-positive cells after 2 consecutive Ctx injuries compared to *Myf5*^*+/+*^ mice (Fig. 3j). A very similar result was obtained upon calculation of the number of Pax7-positive cells per area (*Myf5*^*+/+*^: 18.09 ± 1.02; *Myf5*^*+/Cre*^: 15.57 ± 1.04 Pax7^+^ cells/mm^2^; mean ± SEM; *n* ≥ 4; *p* > 0.05). In summary, these results indicate that mTORC2 signaling may play a role in replenishing the satellite cell pool upon repeated injuries.

### Precocious loss of MuSCs in RImyfKO mice

**Fig. 4:**
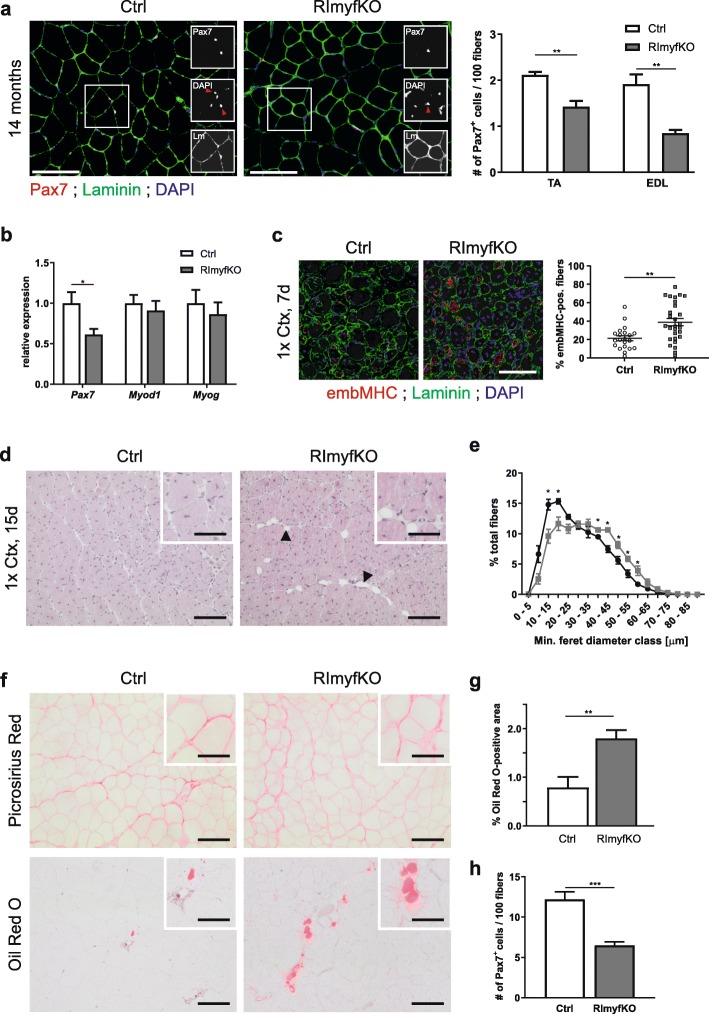
The number of MuSCs and the regenerative capacity of muscle are diminished in 1-year-old RImyfKO mice. **a** Merged and single (insets) staining against Pax7 (red), laminin (green), and for DAPI (blue) of muscle cross-sections from 14-month-old Ctrl and RImyfKO mice. Red arrowheads in insets indicate DAPI-positive nuclei that are also Pax7-positive. Right panel: Quantification of the number of Pax7-positive cells/100 myofibers (*n* = 3–5). Scale bar, 100 μm. **b** Relative expression of transcripts encoding Pax7, MyoD, and MyoG in TA muscle from 14-month-old Ctrl and RImyfKO mice, normalized to *Gapdh* (*n* = 6). **c**–**h** Regenerative response of 1-year-old Ctrl and RImyfKO mice. **c** Cross-sections of TA muscle of 12-month-old Ctrl and RImyfKO mice, 7 days after Ctx injury (1x Ctx, 7 days), stained with antibodies against embMHC (red), laminin (green), and for DAPI (blue). Scale bar, 100 μm. Right panel: Percentage of embMHC-positive fibers (21 or 28 images; *n* = 3 control and 4 RImyfKO mice). **d** H&E coloration of TA muscle cross-sections of Ctrl and RImyfKO mice 15 days after Ctx injury of TA (1x Ctx, 15 days). Arrowheads point to the presence of adipocytes in regenerating RImyfKO muscle. Scale bar, 100 μm and 50 μm (inset). **e** Fiber size distribution of TA muscle, 15-day post-injury (*n* = 3). Grey line: Ctrl; black line: RImyfKO. **f** Picrosirius red coloration of TA muscle 15-day post-injury (top). Oil Red O staining of lipid droplets in TA muscle, 15-day post-injury (bottom). Scale bar, 100 μm and 50 μm (insets). **g** Quantification of relative area positive for Oil Red O staining (*n* = 5). **h** Quantification of the number of Pax7-positive cells/100 myofibers, 15 days after Ctx injury (*n* = 5). All data represent mean ± SEM. *p < 0.05, **p < 0.01, Student’s *t* test

The lower number of Pax7-positive cells after repeated injuries in RImyfKO mice suggests that mTORC2 signaling may contribute to the maintenance of MuSCs. Interestingly, the number of Pax7-positive MuSCs was also significantly reduced in TA and EDL muscles from 14-month-old RImyfKO mice compared to controls (Fig. 4a). Concomitantly, transcript levels encoding Pax7 were lower than in control littermates (Fig. 4b). No difference in the expression levels encoding transcripts for MyoD or MyoG was seen (Fig. 4b). To test whether the lower number of MuSCs resulted in a regeneration deficit, we induced muscle degeneration by Ctx and analyzed muscles 7- and 15-day post injuries. Seven days post injury, RImyfKO mice showed some delay in regeneration as indicated by the higher number of embryonic myosin heavy chain (embMHC)-positive muscle fibers compared to control mice (Fig. 4c). Muscle regeneration was still affected 15 days post injury, indicated by the accumulation of lipid droplets (Fig. 4d), and the shift of the fiber size distribution to smaller fibers (Fig. 4e). While there was no increase in collagen-rich, fibrotic area in regenerating RImyfKO muscle compared to controls (Fig. 4f), lipid infiltration, as visualized by Oil Red O staining, was significantly increased (Fig. 4f, g). Besides this lower regeneration efficacy, the replenishment of the satellite cell pool was also reduced in RImyfKO muscle measured per 100 fibers (Fig. 4h) or number of Pax7-positive cells per area (Ctrl, 36.23 ± 3.47; RImyfKO, 23.84 ± 2.07 Pax7^+^ cells/mm^2^; mean ± SEM; *n* = 4; *p* = 0.022). Thus, mTORC2 signaling in muscle-resident cells contributes to the maintenance of MuSC pool and its depletion affects the regenerative capacity of the muscle at older age.

### mTORC2 signaling does not affect proliferation and self-renewal of MUSCs

**Fig. 5:**
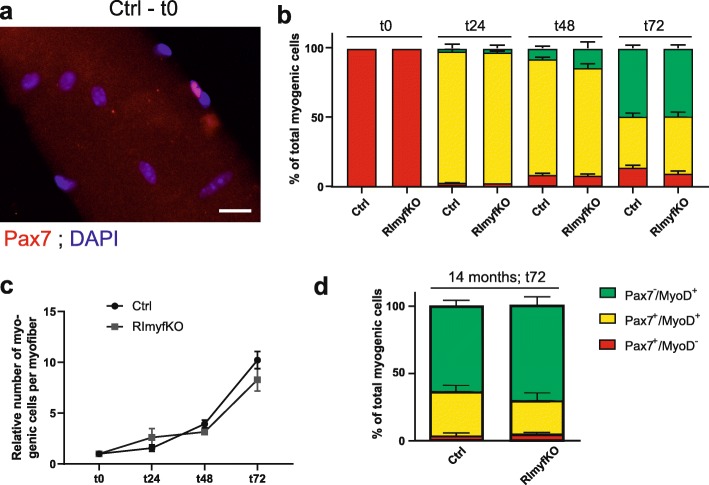
mTORC2 signaling does not affect MuSC quiescence and differentiation. **a**–**d** Single EDL myofibers were isolated from 5-month- (**a**–**c**) or 14-month-old (**d**) control and RImyfKO mice and cultured for different time points. **a** Immunostaining against Pax7 (red) and DAPI staining (blue). Scale bar, 20 μm. **b** Relative number of Pax7- and MyoD-positive cells at time point zero (t0), after 24 h (t24), 48 h (t48), and 72 h (t72) in culture. **c** Mean number of myogenic cells per myofiber at the different time points, normalized to t0. **d** Relative number of Pax7- and MyoD-positive cells at t72 in myofibers isolated from 14-month-old mice. Data represent mean ± SEM (*n* = 3–4 at t0, 3 at t24, 10–11 at t48, and 7 at t72; 20–40 myofibers per animal). Statistics used Student’s *t* test

Loss of MuSCs upon injury may arise from a reduced capacity of the cells to self-renew after activation. To address this, we isolated single fibers from EDL muscle of 5-month-old mice and cultured them under growth-stimulating conditions for up to 72 h. In freshly isolated fibers (time zero; t0), all the cells were Pax7-positive but MyoD-negative (Fig. 5a, b). After 24 h in culture (t24), 95% of the Pax7-positive cells became activated and co-expressed MyoD in both control and RImyfKO cultures (Fig. 5b). There was also no difference in the relative proportion of myogenic cells (i.e., Pax7-negative, MyoD-positive cells; Pax7-positive, MyoD-positive cells; and Pax7-positive, MyoD-negative) in cultured myofibers from RImyfKO or control mice at later time points (Fig. 5b). Finally, the proliferative capacity of RImyfKO and control satellite cells was the same (Fig. 5c). Thus, rictor-deficiency does not affect activation, commitment, and self-renewal of MuSCs in the single muscle fiber preparation. Interestingly, MuSCs on myofibers isolated from 14-month-old RImyfKO mice showed also no difference in their commitment and self-renewal capacity compared to controls (Fig. 5d). Altogether, these results indicate that the reduced number of MuSCs in RImyfKO muscle is not caused by intrinsic differences in proliferation, commitment, and self-renewal.

### Deletion of *Rictor* in myofibers affects the maintenance of MuSCs

**Fig. 6:**
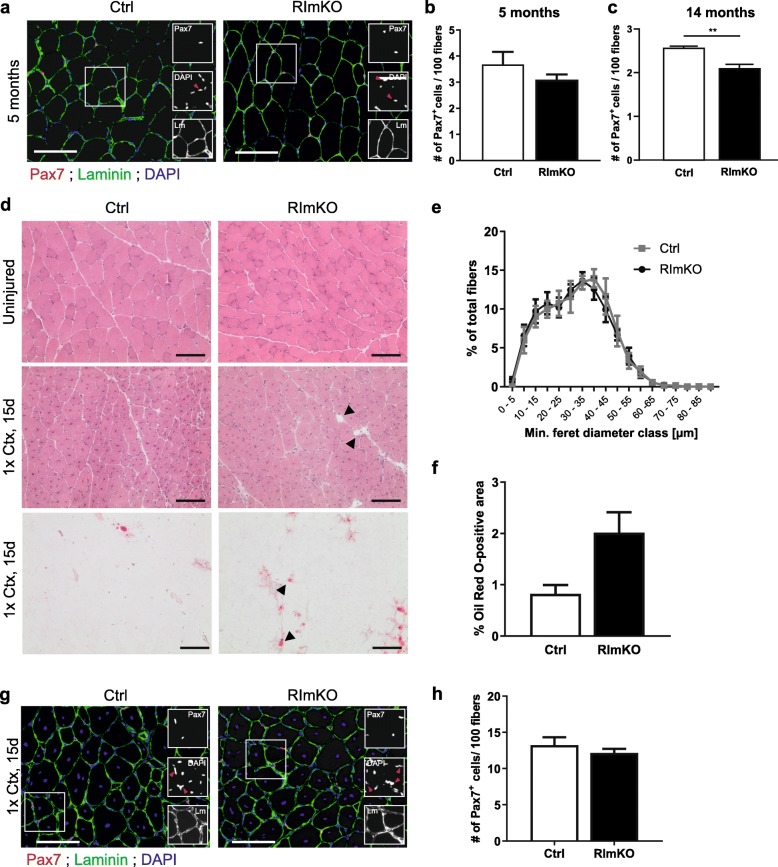
mTORC2 signaling in myofibers affects the maintenance of MuSCs. **a** Merged and single (insets) staining against Pax7 (red), laminin (green), and for DAPI (blue) of muscle cross-sections from 5-month-old Ctrl and RImKO mice. Red arrowheads in insets indicate DAPI-positive nuclei that are also Pax7-positive. **b** Quantification of the number of Pax7-positive cells per 100 myofibers in TA muscle from 5-month-old Ctrl and RImKO mice (*n* = 4–6). **c** Quantification of the number of Pax7-positive cells from 14-month-old Ctrl and RImKO mice (*n* = 3–4). (**d**–**h**) Analysis of regenerating TA muscle of 14-month-old control and RImKO mice, 15 days after cardiotoxin (Ctx)-induced injury (1x Ctx, 15 days). **d** H&E (top) and Oil Red O (bottom) coloration of TA muscle cross-sections. Arrowheads point to adipocytes in regenerating RImKO muscle. Scale bar, 100 μm. **e** Fiber size distribution of TA muscle (*n* = 3-4). **f** Quantification of relative area positive for Oil Red O staining (*n* = 3–4). **g** Merged and single (insets) staining against Pax7 (red), laminin (green) and for DAPI (blue) of muscle cross-sections from 14-month-old Ctrl and RImKO mice, 15 days after Ctx injury. Red arrowheads in insets indicate DAPI-positive nuclei that are also Pax7-positive. **h** Quantification of the number of Pax7-positive satellite cells 15 days post Ctx injury (*n* = 3–4). Data represent mean ± SEM. **p < 0.01, Student’s *t* test (difference between genotypes)

To test whether MuSC numbers were also affected by rictor-dependent, but cell-extrinsic factors, we analyzed the MuSC pool in RImKO mice. In these mice, Cre is driven by the human skeletal actin promoter, which is active in muscle fibers but not in MuSCs [23]. As observed in RImyfKO mice, there was no significant difference in the number of Pax7-positive cells in RImKO (HSA^+*/Cre*^; *Rictor*^*fl/fl*^) muscle compared to controls (HSA^*+/+*^; *Rictor*^*fl/fl*^) at the age of 5 months (Fig. 6a, b). At the age of 14 months, the number of MuSCs was significantly reduced in RImKO muscle compared to controls (Fig. 6c), mirroring the result obtained in RImyfKO mice. These results suggest that mTORC2-dependent, cell-extrinsic factors may contribute to MuSC maintenance. Thus, we next examined the regenerative response to Ctx-induced injury in 14-month-old RImKO mice and analyzed the muscles 15-day post-injury. Myofiber restoration in RImKO mice seemed efficient as indicated by H&E staining and the fiber size distribution (Fig. 6d, e). We also observed some lipid-like structures (Fig. 6d, arrowheads) and lipid infiltration as documented by Oil Red O staining (Fig. 6d). Indeed, the Oil Red O-positive area tended to be larger in RImKO mice than in controls (Fig. 6f). Besides, the number of MuSCs was not significantly reduced after Ctx-induced injury (Fig. 6g, h). The result for the number of Pax7-positive cells per area was also not significant (Ctrl, 36.60 ± 6.91; RImyfKO, 36.93 ± 1.31 Pax7^+^ cells/mm^2^; mean ± SEM; *n* ≥ 3; *p* > 0.05). Together, these results indicate that mTORC2-dependent extrinsic factors contribute to the loss of MuSCs seen in 14-month-old RImyfKO mice.

## Discussion

In this study, we analyzed the function of the mTORC2 pathway in myogenesis by characterizing the consequences of the depletion of rictor, an essential component of mTORC2. In contrast to previous studies in which rictor was depleted from differentiated myofibers [11–13], we used mice that drive Cre expression under the *Myf5* promoter. In the resulting RImyfKO mice, rictor is depleted in the majority of the muscle progenitors [17], in most MuSCs [3] and in myofibers. Thus, RImyfKO mice allowed us to study the role of mTORC2 in embryonic myogenesis and in injury-induced muscle regeneration in the adult.

As recently reported [16] and now confirmed until the age of 5 months, RImyfKO mice did not show any gross abnormalities during the development of skeletal muscle. Moreover, rictor-deficient myoblasts isolated from the mice showed normal proliferation and differentiation capability in culture [16]. We now complement these data using the single-muscle-fiber preparation. In this in vitro assay, MuSC activation, proliferation, and differentiation are identical in RImyfKO and control fibers. These results are important findings as others have reported that the knock-down of *Rictor* in C2C12 myoblasts results in a strong impairment in myoblast differentiation [14]. Thus, C2C12 myoblasts seem to respond differently than primary myoblasts in culture or MuSCs residing in their muscle fiber niche.

Another phenotype of RImyfKO mice was their reduced body and muscle weight compared to controls. As this phenotype is not observed in RImKO mice [11–13], these differences may derive from the *Myf5*-Cre-driven depletion of rictor during muscle development. During development, insulin-like growth factors (IGFs), more specifically IGF-II, have been shown to affect growth [24]. Consistently, *Igf2*-deficient mice and mice carrying a paternal *Igf2* mutation are smaller and show a strong reduction in body weight [25]. As IGF-II translation is controlled by mTOR through the phosphorylation of IGF2 mRNA-binding protein 1 (IMP1) [26], an early impairment of IGF-II production may contribute to the loss of body weight in RImyfKO mice.

The most striking phenotype observed in RImyfKO mice was the reduction in the number of MuSCs after two rounds of Ctx-induced muscle regeneration and the lower number of MuSCs in 14-month-old RImyfKO mice. Consequently, the regenerative capacity of RImyfKO muscle decreased upon injury at this age. We confirmed that the 50% Myf5 reduction (because the Cre cDNA is knocked into the *Myf5* locus) did not cause this lowering of the number of MuSCs. In fact, haplo-insufficiency of *Myf5* has been reported to prime satellite cells for myogenic commitment and to result in a higher self-renewal capacity upon regeneration [27]. These are two events that would cause a phenotype opposite to that observed in RImyfKO mice. In summary, the results of our work indicate that mTORC2 is involved in the maintenance of MuSCs. As there was no change in the expression of myogenic markers in muscle lysates of RImyfKO mice, it is unlikely that the loss of MuSCs arises from self-activation of the cells [28, 29].

We also tested whether mTORC2-dependent, MuSC-extrinsic factors could contribute to the phenotype by examining RImKO mice. The number of MuSCs was indeed lower in 14-month-old RImKO mice compared to controls. However, muscle regeneration was not significantly affected suggesting a minor contribution of cell-non-autonomous factors. One possibility is that such factors are localized in the basal lamina of the MuSC niche. Specific components of this niche, such as fibronectin [30] or collagen V [31], have been implicated in satellite cell maintenance, aging, and the response to injury [32]. Similarly, proteins expressed on the apical side of MuSCs and in muscle fibers affect satellite cell number [32]. Hence, mTORC2-dependent signaling in muscle fibers may affect the MuSC niche.

MuSC fate is also determined by intrinsic signaling pathways. In particular, the conversion of quiescent satellite cells into senescence involves both extrinsic and intrinsic factors [33]. The finding that MuSCs of RImKO mice are less affected than in RImyfKO mice suggests that cell-autonomous mechanisms strongly contribute to the depletion of MuSCs in RImyfKO muscle. The main mTORC2 targets are PKB/Akt [8], PKCα [7], and SGK1 [5]. Although we did not explore which of these targets may affect MuSC function directly, all of them have been implicated in muscle homeostasis and the growth-response of skeletal muscle to external challenges [34–37]. In addition, activation of PKB/Akt by depletion of PTEN in MuSCs causes spontaneous activation of the cells and their premature differentiation [38], which in turn, results in the depletion of the MuSC pool with age [38, 39]. Thus, a mixture of cell-intrinsic and -extrinsic factors, both dependent on mTORC2, may drive the MuSC defects documented here.

## Conclusions

Our study focused on the role of mTORC2 in skeletal muscle fibers and MuSCs. We find that mTORC2 signaling is dispensable for embryonic myogenesis and muscle fiber homeostasis in the adult. In contrast, MuSC maintenance and, as a consequence of the lower number of MuSCs, muscle regeneration at higher age are impaired in the absence of mTORC2. MuSC maintenance is, at least in part, hampered because of alterations in mTORC2-dependent extrinsic signals. These observations provide a framework to the better understanding of mTORC2 signaling in muscle stem cell homeostasis.

## Data Availability

All data generated and analyzed during the study are available from the corresponding author on a reasonable request.

## Abbreviations

Ctx: Cardiotoxin
E: Embryonic day
EDL: Extensor digitorum longus
Gastro: Gastrocnemius
H&E: Hematoxylin and eosin
MHC: Myosin heavy chain
MuSC: Muscle stem cell
PFA: Paraformaldehyde
Sol: Soleus
TA: Tibialis anterior
